# Evaluation of psychometric properties of the eating restriction questionnaire and food involvement inventory in Iranian adults

**DOI:** 10.1038/s41598-023-39885-x

**Published:** 2023-08-20

**Authors:** Kiyana Saadati, Khadije Jahangasht Ghoozlu, Fakhreddin Chaboksavar, Abbas Shamsalinia, Mohammad Reza Kordbageri, Reza Ghadimi, Shabnam Parvizi, Fatemeh Ghaffari

**Affiliations:** 1 Mazandaran University of Medical Sciences, Sari, Islamic Republic of Iran; 2 Nursing Care Research Center, Health Research Institute, Babol University of Medical Sciences, Babol, Islamic Republic of Iran; 3 Shahid Beheshti University (SBU), Tehran, Islamic Republic of Iran; 4 Social Determinants of Health Research center, Health Research Institute, Babol University of Medical Sciences, Babol, Islamic Republic of Iran; 5 Babol University of Medical Sciences, Babol, Islamic Republic of Iran

**Keywords:** Health care, Medical research, Risk factors

## Abstract

The objectives of this study were to translate and validate the Persian version of the food involvement inventory (FII) and eating restriction questionnaire (ERQ) and to determine the measurement invariance based on gender, body mass index (BMI) status, and age. This cross-sectional study included 1100 Iranian adults. Exploratory factor analysis (EFA) and confirmatory factor analysis (CFA) were used to evaluate the construct validity of FII and ERQ. Convergent and discriminant validity, measurement invariance in gender, BMI and age, reliability including internal consistency, and stability were investigated for FII and ERQ. The results showed that the four-factor construct of the FII and the one-factor construct of the ERQ were 44.27% and 55.12% of the total variance, respectively. The factor loadings of all items were > .3 in both scales and none of the items were deleted. Fitting indices indicated that the four-factor construct of the FII and the one-factor construct of the ERQ had a good and acceptable fit among the Iranian adults. The Persian versions of the FII and ERQ, translated into Persian and localized according to international standards, had high construct, convergent and discriminant validity as well as high reliability.

## Introduction

Malnutrition results from the lack of uptake or intake of nutrition and is very common in adults^1^. Malnutrition can lead to changes in body composition (decreased body mass and cell mass), reduced physical, mental and occupational function as well as impaired treatment outcomes in various chronic diseases^2^.

The occurrence of malnutrition depends on various physical, psychological, economic and social conditions and lifestyle^3^. One of the aspects of lifestyle is the beliefs, habits and behaviors in food selection and consumption^4^. Eating restriction (ER) refers to the intentional restriction of caloric intake for weight loss^5^; it is a common practice among young people^6^ and can be a beneficial self-regulatory behavior or have a detrimental effect on health. Galinski et al. found that restricting consumption of sugars, high-fat foods, fats, and starches may predict unhealthy dietary patterns in the population^7^. Restrictive eating behaviors can lead to eating disorders, including anorexia nervosa, bulimia nervosa, and binge eating disorder^8^. However, Jezewska-Zychowicz et al. indicated that limiting food intake and undesirable foods (sugars, sweets, fats) was associated with healthy eating patterns. Participants who limited their total food intake, sugar and/or sweets, and fatty foods adhered to healthy dietary patterns, which may support the positive effects of ER^6^.

Although the goal of ER programs is to benefit adults, this change may affect the quantity and quality of nutrients they receive. Sometimes, it may not be in line with what adults want to eat and may lead to their refusal to eat^9^. Food involvement (FI) is another aspect of lifestyle that plays an important role in the adoption of healthy and unhealthy eating habits^10^.

Rozin et al. described FI as a way of thinking about food, beliefs and behaviors related to diet and health, food concerns, role of food as a positive force in life and satisfaction with the perceived healthiness of a diet^11^. Bell and Marshall defined FI as the degree of importance of food in one’s life^12^, greater attention to food^13^, and a close relationship with hedonic and anhedonic eating^14^. The FI is a personality trait that affects food perception and consumer behavior^10^. Consumers’ involvement with a food product directly or indirectly influences their attitude, knowledge, and evaluation of that food^15^. The FI may be an appropriate measure for distinguishing consumer groups and understanding their food perceptions. Due to the high consumption of food and the diversity and progress of the food industry, it is necessary to investigate the consumers’ food involvement^16^.

Since ER and FI reflect the recent lifestyle of individuals^6^, capturing them through valid and reliable instruments can lead to evidence-based measures. To measure the ER and FI constructs, two instruments, including the eating restriction questionnaire (ERQ)^6^ and the food involvement inventory (FII)^10^ are available. These scales can provide valid and generalizable findings when they are consistent with the culture and individual characteristics of communities. The increase in diverse populations worldwide and the need for cross-cultural and multinational research indicate the need for researchers to have access to reliable and valid instruments or measures that have been cross-validated in different cultural segments of the population and/or in other languages^16^. The psychometric properties of these instruments have not been studied in Iranian adults The results of the studies^17, 18^ show that the rate of obesity and overweight among adults is high in Mazandaran province of Iran. The results of the study by Djalalinia et al. show a geographic pattern at the provincial level in which BMI in the population increases from the southeastern to the northwestern regions of the country. One of the reasons for this is the food culture, i.e. the particular dietary habits and patterns of people in the northern regions of Iran^17^. Variables such as ER and FI reflect a person’s dietary behaviors, habits, beliefs, and attitudes, which can be measured using valid and reliable instruments to identify dietary patterns and malnutrition among adults. In the systems providing health services in Iran, lifestyle screening of adults in different dimensions is done by instruments and is part of the health policies. However, less attention has been paid to the aspect of nutrition and food patterns. One of the reasons is the lack of access to instruments compatible with Iranian culture. FFI and ERQ are instruments that, due to their simplicity and few items, make it possible to use them to provide health services, so we conducted this study with the objectives of the present study were to 1- evaluate the psychometric properties of the Persian version of FII and ERQ in Iranian adults and 2- determine the measurement invariance of FII and ERQ based on gender, body mass index (BMI) status (adults with and without overweight) and age (< 35 years and > 35 years).

## Methods

### Design

The aim of this methodological study (2022) was to translate and validate the Persian versions of FII and ERQ in Iranian adults. The study population included all adults attending comprehensive health service centers in western Mazandaran, Iran. This study was approved with code No. 724133802.

### Instruments

#### Demographic characteristics questionnaire

This questionnaire includes age, gender, educational level, marital status, residence, occupational status, economic status, weight, height, and BMI.

#### Eating restriction questionnaire (ERQ)

This ten-item questionnaire developed by Jezewska-Zychowicz et al. (2020), contains restrictions on the amount of food, cereals, bread, potatoes, sugar or sweets, fats, high-fat foods, meats, dairy products, fish, raw fruits and vegetables^6^. In this study, the ERQ was developed by reviewing texts and interviewing 10 adults who met the inclusion criteria, and three items were added to the questionnaire: “I have eating restriction on nuts (walnuts/peanuts/almonds/pistachios/hazelnuts),” “I have eating restriction on cereals”, and “I have eating restriction on birds’ eggs (chicken or other birds' eggs)”.

#### Food involvement inventory (FII)

The FII designed and psychometrized by Lee et al. (2019) reflects an individual’s current food lifestyle and includes 25 items, and dimensions of affective, cognitive, behavioral-purchase, and behavioral-cooking attitudes. This instrument was scored based on a 5-point Likert scale ranging from 1 to 9 (strongly disagree = 1, disagree = 3, neither disagree nor agree = 5, agree = 7, and strongly agree = 9)^10^.

### Translation process

In developing the Persian version of the FII and ERQ based on the WHO protocol (2015), the forward–backward translation technique was used^19^. In the present study, the translation process was as follows: (a) permission from the instrument designer, (b) forward translation from English (original language) into Persian (target language), (c) reconciliation and matching of forward translations, and (d) backward translation from Persian (target language) into English (original language). Then, instruments’ psychometric properties (validity and reliability) were examined in adults.

### Validity

The current study examined face, content, construct, convergent, and discriminant validity.

#### Face validity

Face validity was determined qualitatively and quantitatively. To quantitatively evaluate face validity, the instruments were given to ten members of the target group and they were asked to comment on the levels of difficulty, irrelevancy and ambiguity of each item through individual and face-to-face interviews. To quantitatively determine face validity, the impact item was calculated using the following formula:

$${\text{Impact item}} = {\text{frequency }}\left( \% \right) \, \times {\text{ importance}}.$$

Impact item ≥ 1.5 remained in the study^20^.

#### Content validity

Content validity was determined qualitatively and quantitatively. To quantitatively assess content validity, ten experts (experienced in qualitative research and instrument development) assessed grammar, wording, item allocation, and scaling of tools.

To quantitatively determine content validity, the content validity ratio (CVR) and content validity index (CVI)^21^ were evaluated. The same experts gave their opinion on each item using the following formula:

$$\mathrm{CVR}=\frac{\mathrm{ne}-\left(\frac{\mathrm{N}}{2}\right)}{\left(\frac{\mathrm{N}}{2}\right)},$$

Ne = The number of experts who selected “necessary”, N = The total number of experts.

The minimum acceptable CVR was determined based on the Lawashe table^22^. The number of experts was 10, so the acceptable value of CVR was ≥ 0.62. The method of Waltz and Bausell ^23^ was used to test the CVI. The same experts determined the relevance of each item and calculated the CVI using the following formula:

$$\mathrm{CVI}=\frac{\mathrm{The\,\, number \,\,of \,\,the \,\,specialists\,\, who\,\, have\,\, checked\,\, option }3\mathrm{ and }4 }{\mathrm{The\,\, total\,\, number\,\, of \,\,specialists}}.$$

Items with a CVI > 0.79 were accepted, whereas items with a CVI of 0.70–0.79 were revised^23^.

#### Construct validity

Construct validity was assessed through exploratory factor analysis (EFA), confirmatory factor analysis (CFA) as well as convergent and discriminant validity. A cross-sectional study was performed to evaluate the construct validity. Samples were selected using the convenience sampling method. Inclusion criteria were 18–60-year-old persons having literacy, as well as exclusion criteria included not completing the questionnaires completely. After measuring BMI, the research instruments were completed by the questioners and with valid instruments by the sample population in the presence of the researcher and given to the researcher. The presence of the researchers during the distribution of the questionnaires, the explanation of the accuracy in the completion of the questionnaires, the explanation of the research objectives, the maintenance of the confidentiality of the participants' information, and the validity of the instrument used to measure height and weight assured us of the quality and accuracy of the information collected. To measure the validation of ERQ and FII constructs, all samples (N = 1100) were randomly divided into two subgroups of 550 individuals. The first subgroup included 302 women and 248 men (M_age_ = 37.73, SD = 11.56; M_BMI_ = 26.77, SD = 6.36), and the second subgroup consisted of 262 women and 282 men (M_age_ = 37.53, SD = 10.62; M_BMI_ = 27.35, SD = 4.76). Exploratory factor analysis (EFA) and confirmatory factor analysis (CFA) were conducted to assess construct validity.

### EFA

The EFA was performed on the first group samples (N = 550) for the FII and ER. The Kaiser–Meyer–Olkin Measure (KMO) and Bartlett’s sphericity tests were applied to assess sample adequacy and sphericity, respectively. Then, the latent factors of both instruments were extracted using the principal axis factoring (PAF) method, varimax rotation, and scree plot. The presence of a single item in the factor was approximately 0.3 based on the following formula:

$$\mathrm{CV}=5.152 \div \sqrt{(n-2)} C.$$

The CV is the number of extractable factors and n is the sample size of the study^24^.

### CFA

CFA of the two instruments was conducted on the samples of the second group (N = 550). Model fitting was carried out using the goodness of fit indices (GFI), Satorra–Bentler scaled chi-square test (S–B χ^2^), comparative fit index (CFI), Tucker–Lewis index (TLI), standardized root mean square residual (SRMR), root mean square error of approximation (RMSEA), and confidence interval (CI) of 90%^14^.

### Convergent and discriminant validity

The correlation between the FII and ERQ, the correlation between the subscales, and the correlation between age, gender and BMI status with the scales and subscales were investigated.

### Measurement invariance

Measurement invariance means that a measurement instrument measures the same psychological structures in different societies. In the current study, measurement invariance in gender, BMI status and age was evaluated based on multigroup CFA using MPlus 6.1 software^25^. The first stage of configural invariance was investigated by assuming the equivalence of model factors among groups, and other models were compared with configural invariance. In the next step, metric invariance was evaluated by assuming equivalence of factor loadings among groups. In the third stage, scalar invariance was assessed by assuming the equivalence of factor loadings and item intercepts among groups. Moreover, the ΔCFI index (CFI change between two nested models) was used as a model equivalence index.

### Reliability

Internal consistency was evaluated based on the Cronbach’s alpha coefficient, McDonald’s omega (Ω) and average inter-item correlation (AIC). The intraclass correlation method was used to evaluate the stability. In this method, 40 individuals who were randomly selected from all initial participants completed the FII and ERQ for the second time in a two-week interval.

### Statistical analysis

In the present study, to evaluate the EFA, the R4.5 software with the Psych and Polycor packages was used for the FII and ERQ, respectively. MPlus6.1 software was used to evaluate the CFA and invariance (configural, metric, and scalar) of the instruments. Convergent and discriminant validity was tested with SPSS 24 using the Pearson correlation test. In addition, the receiver operating characteristic (ROC) curve was utilized to determine the cut-off point.

### Ethical aspects

The current study was approved by the Ethics Committee of Babol University of Medical Sciences (IR.MUBABOL.HRI.REC.1400.046). All methods were carried out in accordance with the relevant guidelines and regulations. The objectives of the ongoing study were explained to all participants and informed written consent was obtained from them. Participant's right to withdraw from the research and maintain confidentiality was observed.

## Results

### Sample characteristics

The results showed that the mean age of participants (51.8% = females, 48.2% = males) was 37.63 ± 6.68 years as well as 56.2 and 43.8% of them were married and single, respectively. Among them, 43.7 and 56.3% were employed and unemployed as well as 42.2 and 57.8% had diploma and academic degrees, respectively. The economic status of 73.5 and 26.5% was sufficient and insufficient, respectively. Totally, 76.7 and 23.3% of them lived in urban and rural areas. The mean weight, height and BMI of the samples were 77.90 ± 13.23 kg, 168.99 ± 9.04 cm and 27.35 ± 4.70, respectively.

### Face and content validity

No items were deleted when studying face validity qualitatively but the statement “When I buy food, I check the information on the package” was deleted when evaluating face validity quantitatively because its value was less than 1.5. The appearance of the items was changed when studying face validity qualitatively. This scale was evaluated by experts for content validity. The CVR and CVI of all samples were acceptable. No item was deleted in this section.

### Construct validity

#### EFA of FII

The results of KMO (0.928) and Bartlett’s sphericity test (5504.831) (P < 0.001) indicated that the data were suitable for EFA. Four factors with eigenvalues > 1 were identified for the FII construct and confirmed based on the screen plot. The data were rotated by varimax rotation, and the four-factor construct of the FII accounted for 44.27% of the total variance. Totally, the first 8-item factor “behavioral-cooking attitude”, the second 6-item factor “affective attitude”, the third 5-item “behavioral-purchase attitude” and the fourth 5-item “cognitive attitude” accounted for 13.01% (eigenvalue = 3.12), 12.75% (Eigenvalue = 3.05), 10.07% (eigenvalue = 2.41) and 8.44% (eigenvalue = 2.03) of the total variance, respectively. Additionally, there was a weak correlation (< 0.3) between the four factors. The findings revealed that factor loadings of all items were > 0.3 and no items were deleted. The correlation between all items and the total score was higher than the minimum acceptable value of 0.3 (Table 1).

**Table 1:** Descriptive statistics, exploratory and confirmatory factor analyses of the FII.

Items	Total sample, N = 1100, means (SD)	EFA sample 1, N = 550				CFA sample 2, N = 550, λ_X_
		F1-L	F2-L	F3-L	F4-L	
Behavioral-cooking	20.76 (4.75)					
I like cooking	2.64 (0.88)	0.525				0.705
I often prepare food and share it with people	2.34 (0.92)	0.377				0.319
I have knowledge about food and cooking	2.62 (0.85)	0.509				0.584
I enjoy food-related TV programs	2.60 (0.92)	0.530				0.593
After eating delicious food elsewhere, I make it myself	2.56 (0.90)	0.528				0.563
I enjoy food-related information on SNS and blogs	2.60 (0.94)	0.646				0.557
I enjoy buying and preparing food	2.66 (0.88)	0.627				0.668
I am interested in recipes	2.70 (0.89)	0.585				0.636
Affective	15.88 (3.36)					
I enjoy talking about food	2.69 (0.90)		0.315			0.455
I am very concerned about what I eat	2.43 (0.89)		0.449			0.435
I often think about what I ate or am going to eat	2.67 (0.86)		0.502			0.538
Food gives me pleasure	2.68 (0.90)		0.617			0.526
Food is an important part of my life	2.73 (0.88)		0.560			0.527
I am interested in food	2.66 (0.89)		0.498			0.571
Behavioral-purchase	13.31 (2.97)					
I look for relevant information before purchasing food	2.56 (0.88)			0.409		0.607
I consider many things when I buy food	2.73 (0.88)			0.647		0.557
I look for several retailers (on-line and off-line) before purchasing food	2.43 (0.93)			0.575		0.446
I try to buy satisfactory food	2.80 (0.89)			0.652		0.506
I compare different options before purchasing food	2.77 (0.95)			0.389		0.526
Cognitive	12.77 (2.86)					
I spend much time and effort choosing food	2.06 (0.89)				0.351	0.458
I can recommend certain food items to others	2.69 (0.86)				0.468	0.512
When I choose food, I am confident in my choice	2.72 (0.92)				0.500	0.535
Generally, I can specify the reason why I chose a particular food	2.62 (0.82)				0.414	0.489
I focus on information about food	2.66 (0.91)				0.623	0.577

*EFA* exploratory factor analysis, *CFA* confirmatory factor analysis, *M* means, *SD* standard deviation, *F1*, *F2*, *F3*, *F4*-*L* factor 1, factor 2, factor 3, factor4 – loadings, *λx* standardized coefficients.

#### CFA of FII

Fitting indices demonstrated that the four-factor construct of FII had a good and acceptable fit in the Iranian adult community: S-B χ^2^ = 914.926, DF = 246, P < 0.001, CFI = 0.921, TLI = 0.928, RMSEA = 0.070 (90% C.I 0.065–0.075), SRMR = 0.069. All factor loadings of the FII items were significant on their factors (all PS < 0.001) (Table 1).

### EFA of ERQ

The KMO (0.901) and Bartlett's sphericity test (2339.008) (P < 0.001) illustrated that the data were suitable for EFA. A factor with a eigenvalues of > 1 was identified for the ER construct and confirmed according to the screen plot. The data were rotated by varimax rotation, and in total, one-factor construct of ER allocated 55.12% (Eigenvalue = 7.33) of the total variance. Factor loadings of all items were > 0.4 and no items were deleted. The correlation between all items and the total score was higher than the minimum acceptable value of 0.3 (range = 0.47–0.72, mean = 0.530) (Table 2).

**Table 2:** Descriptive statistics, exploratory and confirmatory factor analyses of the ERQ.

Items	Total sample N = 1100, M(SD)	EFA sample 1 n = 550	CFA sample 2 n = 550, λ_X_
Eating restrictions	3.13 (3.42)		
I have eating restriction on the quantity of food	0.35 (0.47)	0.903	0.563
I have eating restriction on sugar or sweets	0.27(0.44)	0.688	0.688
I have eating restriction on high-fat foods	0.32 (0.47)	0.677	0.497
I have eating restriction on fats (animal and vegetable fats)	0.27 (0.44)	0.659	0.795
I have eating restriction on bread and cereals such as bread, pasta, wheat, barley, corn, rice and potatoes	0.22 (0.41)	0.854	0.676
I have eating restriction on red and white meat	0.21 (0.40)	0.716	0.621
I have eating restriction on seafood such as shrimp and fish	0.23 (0.42)	0.774	0.666
I have eating restriction on dairy products	0.16 (0.36)	0.640	0.687
I have eating restriction on raw vegetables	0.24 (0.42)	0.785	0.738
I have eating restriction on raw fruits	0.19 (0.39)	0.702	0.645
I have eating restriction on the nuts (walnuts/peanuts/almonds/pistachios/ hazelnuts)	0.22 (0.41)	0.791	0.770
I have eating restriction on the consumption of cereals	0.15 (0.36)	0.674	0.893
I have eating restriction on birds’ eggs (chicken or other birds’ eggs)	0.24 (0.43)	0.843	0.502

### CFA of ERQ

Fitting indices displayed that the one-factor construct of ER had a good and acceptable fit in the Iranian adult community (S–B χ^2^ = 142.075, DF = 58, P < 0.001, CFI = 0.941, TLI = 0.921, RMSEA = 0.073 (90% CI 0.058–0.088), SRMR = 0.066.). All factor loadings of the ERQ items were significant on their factors (all PS < 0.001) (Table 2).

### Reliability (internal consistency and stability) and convergent and discriminant validity

The internal consistency values of all samples based on Cronbach's alpha were 0.814, 0.793, 0.767, and 0.754 for the FII subscales, including behavioral-cooking attitude, affective attitude, behavioral-purchase attitude and cognitive attitude as well as the total score of internal consistency of FII and ERQ was 0.786 and 0.865, respectively. The internal consistency values of all samples based on McDonald's omega were 0.811, 0.792, 0.765, and 0.753 for the FII subscales, including behavioral-cooking attitude, affective attitude, behavioral-purchase attitude and cognitive attitude as well as the total score of internal consistency of FII and ERQ was 0.780 and 0.854, respectively.

The results exhibited that the intraclass correlation coefficients (ICC) were 0.95, 0.91, 0.94, and 0.93 for the FII subscales consisting of behavioral-cooking attitude, affective attitude, behavioral-purchase attitude, and cognitive attitude as well as the ICC total score of FII and ERQ was.92 and 0.96, respectively, which were significant at the level of 0.001.

Table 3 shows the correlation between the FII and ERQ and the variables of age, gender and BMI status. There was a moderate to weak correlation (0.205–0.477) between all FII factors. In addition, a positive and significant correlation was found between BMI status, gender, FII and ERQ. Furthermore, a positive and significant correlation was observed between age and ER (r = 0.478) and a negative and significant correlation was seen between age, affective attitude (r = − 0.267), and cognitive attitude (r = − 0.301). There was a significant negative correlation between FII and ERQ.

**Table 3:** Correlations between FII and ERQ with demographic variables (n = 1100).

	Behavioral-cooking	Affective	Behavioral-purchase	Cognitive	Eating restrictions
Behavioral-cooking	–	0.477^b^	0.341^b^	0.205^b^	− 0.513^b^
Affective		–	0.305^b^	0.248^b^	− 0.572^b^
Behavioral-purchase			–	0.331^b^	− 0.495^b^
Cognitive				–	− 0.384^b^
Eating restrictions					
BMI^c^	0.325^b^	0.323^b^	0.417^b^	0.410^b^	0.337^b^
Age	− 0.056	− 0.267^a^	− 0.012	− 0.301^b^	0.478^b^
Sex	0.334^b^	0.239^b^	0.155^b^	0.248^b^	0.301^b^

Pearson correlation or point-biserial correlations.

^a^correlation is significant at the 0.05 level (2-tailed).

^b^Correlation is significant at the 0.01 level (2-tailed).

^c^Polyserial correlations.

### Analysis of measurement invariance based on gender, BMI status, and age

Before analysis of measurement invariance, the fitting status of the four-factor model of the FII and one-factor model of the ERQ was evaluated based on gender, BMI status, and age of the samples. Then, configural, metric, and scalar invariances were assessed based on gender, BMI status, and age. The results revealed that by observing the values of factor loadings and intercept, the CFI change between configural and constrained models (∆CFI) was < 0.01. Thus, the configural, metric and scalar invariances in the FII and ER scales were confirmed based on gender, BMI status, and age (Tables 4 and 5).

**Table 4:** FII: goodness-of-fit indices of the four-factor model and measurement invariance based on gender, BMI status and age.

Model	S-B χ^2^	dF	TLI	SRMR	RMSE (90% CI)	CFI	ΔCFI
Four-factor	914.926	246	0.921	0.069	0.070 (0.065–0.075)	0.928	
Sex							
Men (n = 536)	993.981	246	0.914	0.068	0.075 (0.068–0.082)	0.916	
Women (n = 564)	997.949	246	0.913	0.057	0.074 (0.069–0.078)	0.919	
Multigroup comparisons							
Configural invariance	2291.946	492	0.922	0.068	0.058(0.055–0.060)	0.927	
Metric invariance	2322.311	512	0.923	0.067	0.057 (0.54–0.059)	0.928	− 0.001
Scalar invariance	2359.114	522	0.925	0.067	0.057 (0.054–0.059)	0.928	− 0.001
BMI status							
Normal weight (n = 430)	890.736	246	0.909	0.063	0.078 (0.074–0.082)	0.910	
Overweight (n = 670)	1115.613	246	0.906	0.056	0.073 (0.068–0.077)	0.907	
Multigroup comparisons							
Configural invariance	2206.540	492	0.907	0.063	0.056 (0.054–0.059)	0.913	
Metric invariance	2236.654	512	0.912	0.065	0.055 (0.053–0.058)	0.914	− 0.001
Scalar invariance	2294.933	522	0.910	0.077	0.055 (0.053–0.058)	0.913	0.000
Age							
Younger-old (< 35years) (n = 514)	986.459	246	0.911	0.056	0.077 (0.072–0.082)	0.913	
Oldest-old (> 35years) (n = 586)	1133.844	246	0.906	0.062	0.078 (0.074–0.082)	0.909	
Multigroup comparisons							
Configural invariance	2220.291	492	0.921	0.056	0.057(0.054–0.059)	0.925	
Metric invariance	2256.621	512	0.914	0.059	0.056(0.053–0.058)	0.926	− 0.001
Scalar invariance	2276.675	522	0.914	0.064	0.055(0.053–0.058)	0.926	0.000

*S*–*B χ*^2^ Satorra–Bentler scaled chi-square test, *df* degree of freedoms, *TLI* Tucker–Lewis index, *SRMR* standardized root-mean square residual, *RMSEA 90% CI* root mean square error of approximation RMSEA and its confidence interval, *CFI* comparative fit index, *D* difference values, *CI* configural invariance, *WI* weak (or metric) invariance, *SI* strong (or scalar) invariance.

**Table 5:** ERQ: goodness-of-fit indices of the first-factor model and measurement invariance based on gender, BMI status and age.

Model	S-B χ^2^	dF	TLI	SRMR	RMSEA (90%CI)	CFI	ΔCFI
One-factor	142.075	58	0.921	0.066	0.073 (0.058–0.088)	0.941	
Sex							
Men (n = 536)	149.878	58	0.923	0.058	0.075 (0.060–0.090)	0.943	
Women (n = 564)	152.116	58	0.924	0.057	0.078 (0.063–0.093)	0.943	
Multigroup comparisons							
Configural invariance	337.936	130	0.926	0.052	0.038 (0.032–0.044)	0.933	
Metric invariance	362.597	142	0.924	0.053	0.037 (0.031–0.043)	0.932	0.001
Scalar invariance	396.934	154	0.924	0.052	0.037 (0.031–0.043)	0.932	0.001
BMI-status							
Normal weight (n = 430)	214.439	58	0.957	0.054	0.079 (0.068–0.091)	0.968	
Overweight (n = 670)	285.722	58	0.917	0.053	0.071 (0.065–0.074)	0.938	
Multigroup comparisons							
Configural invariance	315.819	130	0.935	0.063	0.036 (0.030–0.042)	0.942	
Metric invariance	373.273	142	0.931	0.065	0.038 (0.032–0.044)	0.943	− 0.001
Scalar invariance	384.782	154	0.934	0.077	0.036 (0.031–0.042)	0.944	− 0.002
Age							
Younger-old (< 35years) (n = 514)	212.032	58	0.948	0.053	0.071 (0.065–0.075)	0.954	
Oldest-old (> 35years) (n = 586)	266.155	58	0.929	0.051	0.078 (0.062–0.092)	0.941	
Multigroup comparisons							
Configural invariance	310.402	130	0.948	0.061	0.035 (0.029–0.041)	0.953	
Metric invariance	370.271	142	0.947	0.062	0.038 (0.031–0.044)	0.952	0.001
Scalar invariance	381.826	154	0.946	0.069	0.036 (0.030–0.040)	0.952	0.001

*S*–*B χ*^2^ Satorra–Bentler scaled chi-square test, *df* degree of freedoms, *TLI* Tucker–Lewis index, *SRMR* standardized root-mean square residual, *RMSEA 90% CI* root mean square error of approximation RMSEA and its confidence interval, *CFI* comparative fit index, *D* difference values, *CI* configural invariance, *WI* weak (or metric) invariance, *SI* strong (or scalar) invariance.

### Cut-off point of FII

**Figure 1:**
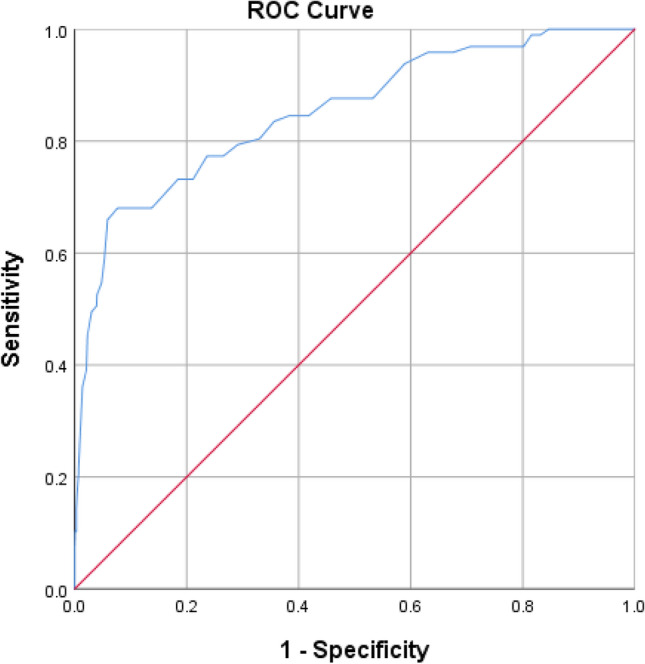
Receiver operating characteristic curve of FII.

A cut-off point of FII was determined by considering the ERQ scale based on the ROC analysis. The area under the ROC curve was 0.851 with a standard error of 0.023 (CI 0.807–0.896) (Fig. 1). According to the ROC curve, the most suitable cut-off point for measuring the FII in adults was 52.50 as well as the sensitivity and specificity were 0.990 and 0.816, respectively.

## Discussion

The aim of the present study was to translate the FII and ERQ scales, to evaluate their psychometric properties and to determine the measurement invariance in terms of age, gender and BMI status. The results of evaluating KMO and Bartlett’s sphericity test indicated the suitability of the data for EFA. Since the KMO ranging from 0.7 to 0.8 and from 0.8 to 0.9 is good and excellent, the KMO values were excellent for both scales of the current study^26^.

The results of the current study showed that the four-factor construct of FII including behavioral- cooking attitude, affective attitude, behavioral-purchase attitude and cognitive attitude accounted for 44.27% of the total variance. This value was 63.32% in the study of Lee et al. (2019)^10^. The results of the present study suggested that there was a weak correlation between the four factors of FII, indicating that this scale had four independent factors. The findings of the ongoing study exhibited that in total, one-factor construct of ER allocated 55.12% of the total variance, and based on the screen plot diagram, it was confirmed that the ER scale was a single factor.

The results demonstrated that the correlation between all items and the total score in both scales was higher than the minimum acceptable value, representing that all items on both scales had inclusion criteria for performing EFA. Based on Cohen’s recommendations, the correlations of 0.20, 0.40 and 0.60 were classified small, moderate and large, respectively^27^.

Since the cut-off points of CFI and TLI > 0.90 as well as SRMR and RMSEA ≤ 0.08 were considered as acceptable and good limit^28^ the fitting indices of the present study showed that the four-factor construct of FII and one-factor construct of ERQ had good and acceptable fit in Iranian adult community.

To evaluate the reliability of the scales, internal consistency was investigated based on Cronbach’s alpha. The results showed that the internal consistency values of all samples in the FII scale, its subscales and ERQ scale were higher than the recommended value of 0.7^29^. On the other hand, considering that in this study, the Ω and α coefficients of the FII and ERQ scales were greater than 0.7 and the AIC values were between 0.2 and 0.4; therefore, the findings represented that both scales had good internal consistency^30^.

The findings of the present study suggested that the ICC for the scale and subscales of FII as well as the ERQ scale was higher than the recommended value of 0.8^29^, illustrating that the two scales had acceptable stability. The FII reliability in the study of Lee et al. (2019) was confirmed based on an overall Cronbach’s alpha coefficient of 0.94 and item discrimination (with corrected item-total correlations greater than 0.40; ranging from 0.44 to 0.77)^10^.

Overall, the results manifested that both scales had appropriate and acceptable convergent and discriminant validity^29^. There was a significant positive correlation between BMI status and gender with FII and ERQ scales, representing that FI and ER were higher in overweight adults than in normal-weight ones and in women than men.

The findings revealed that ER in adults increased with age. Furthermore, there was a significant negative correlation between age with affective attitude and cognitive attitude, meaning that with increasing age, a person’s cognitive and affective attitude decreases. It seems that as people get older, they talk less about food, food is less important for their lives or they spend less time and effort choosing food, which may increase the risk of malnutrition in these people.

Studies suggest that cognitive and affective components play an important role in weight gain or weight loss^31, 32^. For example, people who are overweight or obese eat regardless of their physiological state and are more likely to react to internal situational or emotional factors such as negative mood, fatigue or boredom. These people are more prone to emotional eating, which is detrimental to their self-efficacy and motivation to maintain weight loss over time^33^. Therefore, screening for eating disorders and FI as risk factors for malnutrition in individuals > 35 years old, overweight women and overweight people can help prevent malnutrition in adults.

The results illustrated that there was a significant negative correlation between FII and ERQ. In this way, with increasing FI, ER was reduced. However, the available evidence shows that there is a direct and two-way relationship between ER and FI. For example, the ER on the three foods “fast foods and sweets” and “meat and meat products” was reported more in people with more FI. In addition, people with more FI than individuals with less FI reported a tendency to have more ER^6^.

The results displayed that the CFI change between configural and constrained models (∆CFI) was < 0.01. Hence, the configural, metric and scalar invariances in FII and ER scales were confirmed based on gender, BMI status and age. The ΔCFI ≥ 0.01 indicates a significant decrease in model fit and leads to the rejection of the constrained model^26^. In the ongoing study, the two FII and ERQ scales were invariant and unbiased between groups (gender, BMI status and age). Therefore, the differences between the groups in the scores of the two scales can be interpreted based on subgroups. This means that both FII and ERQ scales can be used with the structure presenting in both groups according to gender, BMI and age.

The strengths of this study are (a) localizing two scales that measure the recent lifestyle of individuals, (b) screening the risk of malnutrition using these two scales, (c) having a large sample size for EFA and CFA, (d) evaluating measurement invariance in terms of gender, BMI status and age, (e) determining the FII cut-off point based on ROC curve and (f) using a weight estimator for CFA to increase the accuracy of the results and the lack of dependence on the assumption of normal data. The use of a convenient sampling method may limit the generalizability of the results. Physical, psychological, social and environmental variables may be effective in ER and FI, a few of which have been addressed in the present study. Both scales were self-reported, which may lead to reporting errors.

## Conclusion

The FII and ERQ were translated into Persian according to international standards; their psychometric properties were studied in Iranian adults that had high construct, convergent, discriminant validity and reliability. Both scales had acceptable validity and reliability for men and women, individuals with normal BMI and overweight, and individuals aged < 35 and > 35 years. The use of these scales due to the small number and simplicity of their items is recommended to measure the recent diet of adults and diagnose the risk of malnutrition.

## Data Availability

The original contributions presented in the study are included in the article/supplementary material, and further inquiries can be directed to the corresponding author.

## References

[1] Cederholm T, Barazzoni R, Austin P, Ballmer P, Biolo G, Bischoff SC, Compher C, Correia I, Higashiguchi T, Holst M (2017). ESPEN guidelines on definitions and terminology of clinical nutrition. Clin. Nutr..

[2] Chen YN, Wall KM, Fofana K, Navarro-Colorado C (2019). Nutrition indicators as potential predictors of AIDS-defining illnesses among ARV-naïve HIV-positive adults in Kapiri Mposhi, Zambia 2008–2009. PLoS One.

[3] Besora-Moreno M, Llauradó E, Tarro L, Solà R (2020). Social and economic factors and malnutrition or the risk of malnutrition in the elderly: A systematic review and meta-analysis of observational studies. Nutrients.

[4] Smith ML, Bergeron CD, Lachenmayr S, Eagle LA, Simon JR (2020). A brief intervention for malnutrition among older adults: Stepping up your nutrition. Int. J. Environ. Res. Public Health.

[5] Herman CP, Mack D (1975). Restrained and unrestrained eating. J. Pers..

[6] Jezewska-Zychowicz M, Gębski J, Kobylińska M (2020). Food involvement, eating restrictions and dietary patterns in polish adults: Expected effects of their relationships (lifestyle study). Nutrients.

[7] Galinski G, Lonnie M, Kowalkowska J, Wadolowska L, Czarnocinska J, Jezewska-Zychowicz M, Babicz-Zielinska E (2016). Self-reported dietary restrictions and dietary patterns in Polish girls: A short research report (GEBaHealth Study). Nutrients.

[8] Elran-Barak R, Sztainer M, Goldschmidt AB, Crow SJ, Peterson CB, Hill LL, Crosby RD, Powers P, Mitchell JE, Le Grange D (2015). Dietary restriction behaviors and binge eating in anorexia nervosa, bulimia nervosa and binge eating disorder: Trans-diagnostic examination of the restraint model. Eat. Behav..

[9] Buckler DA, Kelber ST, Goodwin JS (1994). The use of dietary restrictions in malnourished nursing home patients. J. Am. Geriatr. Soc..

[10] Lee YM, Lee EK, Chung SJ, Kim CY, Kim KO (2020). Development and validation of the food involvement inventory (FII) featuring the attitudinal constructs. Food Sci. Biotechnol..

[11] Rozin P, Fischler C, Imada S, Sarubin A, Wrzesniewski A (1999). Attitudes to food and the role of food in life in the USA, Japan, Flemish Belgium and France: Possible implications for the diet–health debate. Appetite.

[12] Bell R, Marshall DW (2003). The construct of food involvement in behavioral research: Scale development and validation. Appetite.

[13] Barker M, Lawrence W, Woadden J, Crozier SR, Skinner TC (2008). Women of lower educational attainment have lower food involvement and eat less fruit and vegetables. Appetite.

[14] Marshall D, Bell R (2004). Relating the food involvement scale to demographic variables, food choice and other constructs. Food Qual. Prefer..

[15] Ares G, Besio M, Giménez A, Deliza R (2010). Relationship between involvement and functional milk desserts intention to purchase Influence on attitude towards packaging characteristics. Appetite.

[16] Sousa VD, Rojjanasrirat W (2011). Translation, adaptation and validation of instruments or scales for use in cross cultural health care research: A clear and user friendly guideline. J. Eval. Clin. Pract..

[17] Djalalinia S, Saeedi Moghaddam S, Sheidaei A, Rezaei N, Naghibi Iravani SS, Modirian M, Zokaei H, Yoosefi M, Gohari K, Kousha A, Abdi Z (2022). Patterns of obesity and overweight in the Iranian population: Findings of STEPs 2016. Front. Endocrinol..

[18] Ahmadi M, Moosazadeh M, Vardanjani HM, Dehghan A (2014). Prevalence of obesity and overweight and their related factors among the adults of Mazandaran Province, Iran, in 2010. Electron. Physician.

[19] World health organization. Process of translation and adaptation of instruments. World Health Organization. http://www.who.int/substance_abuse/research_tools/translation/en/ (2015).

[20] Hajizadeh E, Asghari M (2011). Statistical Methods and Analyses in Health and Biosciences a Research Methodological Approach.

[21] Almanasreh E, Moles R, Chen TF (2019). Evaluation of methods used for estimating content validity. Res. Soc. Adm. Pharm..

[22] Lawshe CH (1975). A quantitative approach to content validity. Pers. Psychol..

[23] Waltz CF, Bausell BR (1981). Nursing Research: Design Statistics and Computer Analysis.

[24] Fok, D. Development and testing of a low vision product selection instrument (LVPSI): A mixed-methods approach. Electronic Thesis and Dissertation Repository. The University of Western Ontario (2011).

[25] Byrne BM (2013). Structural Equation Modeling with Mplus: Basic Concepts, Applications, and Programming.

[26] Cheung GW, Rensvold RB (2002). Evaluating goodness-of-fit indexes for testing measurement invariance. Struct. Equ. Model..

[27] Cohen J (1992). Statistical power analysis. Curr. Dir.Psychol. Sci..

[28] Kline R, Kline R (2016). Data preparation and psychometrics review. Principles and Practice of Structural Equation Modeling.

[29] Loewenthal K, Lewis CA (2018). An Introduction to Psychological Tests and Scales.

[30] Mohammadbeigi A, Mohammadsalehi N, Aligol M (2015). Validity and reliability of the instruments and types of measurments in health applied researches. J. Rafsanjan Univ. Med. Sci..

[31] Davidson TL, Jones S, Roy M, Stevenson RJ (2019). The cognitive control of eating and body weight: It’s more than what you “think”. Front. Psychol..

[32] Ingels JS, Zizzi S (2018). A qualitative analysis of the role of emotions in different patterns of long-term weight loss. Psychol. Health.

[33] Wehling H, Lusher JM (2019). Cognitive and emotional influences on eating behaviour: A qualitative perspective. Nutr. Metab. Insights.

